# Case Report: Spinal muscular atrophy with IgA nephropathy: a coincidence or association?

**DOI:** 10.3389/fped.2025.1728887

**Published:** 2026-01-22

**Authors:** Yuxuan Gu, Le Wang, Xiaoying Yuan, Yanan Han, Peitong Han, Jieyuan Cui, Xinlei Wang, Yuchan Huang, Lili Zhang, Chunzhen Li

**Affiliations:** 1 Department of Nephrology and Immunology, Hebei Children's Hospital, Shijiazhuang, Hebei, China; 2 Institute of Pediatric Research, Hebei Children's Hospital, Shijiazhuang, Hebei, China; 3 Department of Pathology, Hebei Children's Hospital, Shijiazhuang, Hebei, China; 4 Hebei Key Laboratory of Pediatric Allergy and Immunology, Hebei Children's Hospital, Shijiazhuang, Hebei, China; 5 Hebei Clinical Medicine Research Center for Children's Health and Diseases, Hebei Children’s Hospital, Shijiazhuang, Hebei, China; 6 Mechanism Elucidation and Clinical Intervention of Pediatric Immune-Related Diseases Under Cross-Border Collaboration, Hebei Children's Hospital, Shijiazhuang, Hebei, China

**Keywords:** IgA nephropathy, motor neuron disease, nephrosis, pediatric, spinal muscular atrophy

## Abstract

**Background:**

Spinal muscular atrophy (SMA) is an autosomal recessive neuromuscular disorder caused by biallelic loss-of-function variants of the survival motor neuron 1 (*SMN1*) gene on chromosome 5q13. It has been reported that SMA may affect the function of the kidneys. Here, we report a patient with co-occurrence of SMA and IgA nephropathy (IgAN).

**Case presentation:**

A 14-year-old girl presented with six months of limb weakness, progressive exacerbation of symptoms of left lower limb muscle weakness; her left lower limb muscle strength decreased, and bilateral knee tendon reflexes and Achilles tendon reflexes were not elicited. The patient was diagnosed with SMA type 3 in conjunction with the results of genetic testing. The patient had proteinuria and hematuria, and a renal biopsy was performed. Considering the patient's clinical and pathological characteristics, the final diagnosis was spinal muscular atrophy combined with IgA nephropathy. To the best of our knowledge, this is the first reported case that demonstrates the coexistence of SMA and IgAN.

**Discussion and conclusions:**

The exact mechanism of renal impairment due to SMA is not fully understood, and the combination of SMA with IgAN is extremely rare. Our report suggests that there may be a potential association between them.

## 1 Introduction

SMA is an autosomal recessive neuromuscular disease with an incidence of approximately 1 in 10,000 live births (1). It is characterized by degeneration of motor neurons in the anterior horn of the spinal cord, resulting in muscle weakness and atrophy. IgA nephropathy (IgAN) is the most prevalent primary glomerular disease worldwide, accounting for approximately 10%–50% of biopsy-proven glomerulopathies depending on the region (2). The latest evidence indicates that SMA is a multisystem disease with dysfunction in multiple organ systems, including skeletal muscle, the heart, kidney, and connective tissue. In recent years, cases of SMA combined with renal injury have been documented (3, 4). This article reports a patient with SMA type 3 whose renal biopsy revealed a diagnosis of IgAN. To the best of our knowledge, this is the first reported case that demonstrates the coexistence of SMA and IgAN, and this case serves to offer a reference for a possible association between the two.

## 2 Case presentation

A 14-year-old female was admitted to the Department of Neurology of our hospital on March 30, 2022, owing to “limb weakness for half a year”. The patient had persistent weakness of the left lower limb with no obvious inducement for half a year. She could stand and walk alone but struggled to go upstairs. There was occasional pain below the left knee, which could be relieved by rest. She was not given any special treatment. One month ago, her symptoms worsened. She was still able to stand and walk alone, but she had trouble getting up from a squatting position and could not lift her legs in the supine position. The patient had a slightly abnormal walking posture since childhood, with both feet internally rotated and less movement than children of the same age. She consulted our hospital at the age of 3, and the electromyography (EMG) revealed neurogenic damage. However, owing to the limited availability of genetic testing technology at the time and the family's restricted awareness of the disease, neither further molecular genetic diagnosis was pursued nor long-term follow-up established. The parents were healthy and denied any family history of hereditary or similar diseases. This patient has no history of recurrent infections. Approximately three months prior to admission, a comprehensive physical examination was conducted, with both blood and urine routine tests showing no abnormalities.

The patient showed a favorable mental response, accurate answers, normal development, and no obvious abnormalities on cardiopulmonary or abdominal examination. The patient is overweight, with a height of 162 cm, weight of 64 kg (90–97th percentile), and a BMI of 26.3. The muscle tone of the limbs was normal, and the muscle strength of the left lower limb was grade V—. Bilateral knee tendon reflexes and Achilles tendon reflexes were not elicited, and Gower's sign was positive.

Laboratory studies revealed the following: blood routine, liver and kidney functions, and homocysteine showed no obvious abnormalities; cardiac enzymes: Creatine kinase (CK): 684 U/L (normal range 40–200 U/L); Creatinine kinase-myocardial band (CK-MB): 12.7 ng/mL (normal range <5 ng/mL); EMG: upper and lower limb EMG suggested neurogenic damage; no obvious abnormalities were found via cranial MRI plain scanning. Genetic detection: gene deletion duplication analysis: the copy-number analysis revealed a homozygous deletion of exons 4–5 in the neuronal apoptosis inhibitory protein (*NAIP*) gene, which was classified as pathogenic according to the American College of Medical Genetics and Genomics (ACMG) guidelines. SMA genetic test: SMA genetic testing showed 0 copies of *SMN1* exon 7 and no deletion of *SMN1* exon 8, together with 2 copies of *SMN2* exon 7, confirming 5q-SMA due to biallelic *SMN1* loss. After admission, the following examinations were completed: coagulation routine, electrolytes, sugar, cardiac ultrasound, electrocardiogram, and CT scan of the chest: no significant abnormalities; full spine in front and side view: no obvious abnormality of the spinal bone; the angle of spinal scoliosis is approximately 7°; and lung function: ventilation is normal.

The patient was treated with an intrathecal injection of Nusinersen 12 mg, along with oral coenzyme Q10 and L-carnitine for symptomatic myocardial nutrition. After the treatment, the child's left lower limb weakness slightly improved. At admission and during hospitalization (before the application of Nusinersen), the child was subjected to the following tests: urine routine: protein: 3+∼4+, red blood cells: 31.5∼305.1/HPF (normal range 0–4/HPF), white blood cells: 6.6∼15.0/HPF (normal range 0–5/HPF); four items of premature renal loss: urinary microalbumin: 2,250 mg/L (normal range 0–30 mg/L), N-acetyl β-D glucosaminidase: 18.5 IU/L (normal range <12 IU/L), urinary transferrin: 177 mg/L (normal range 0–2.35 mg/L); serum proteins: total protein: 48.8 g/L (normal range 60–80 g/L), albumin: 28.4 g/L (normal range 35–55 g/L); and urological color ultrasound: bilateral renal cortical echogenicity enhancement. After being transferred to the Nephrology and Immunology Department, she was found to have a urine protein concentration of 3.89 g/24 h; autoantibodies, antinuclear antibodies, antiglomerular basement membrane antibodies, anticardiolipin antibodies, vasculitis, lupus-like anticoagulant screening, and T-cell tests for tuberculosis infection were all negative.

**Figure 1:**
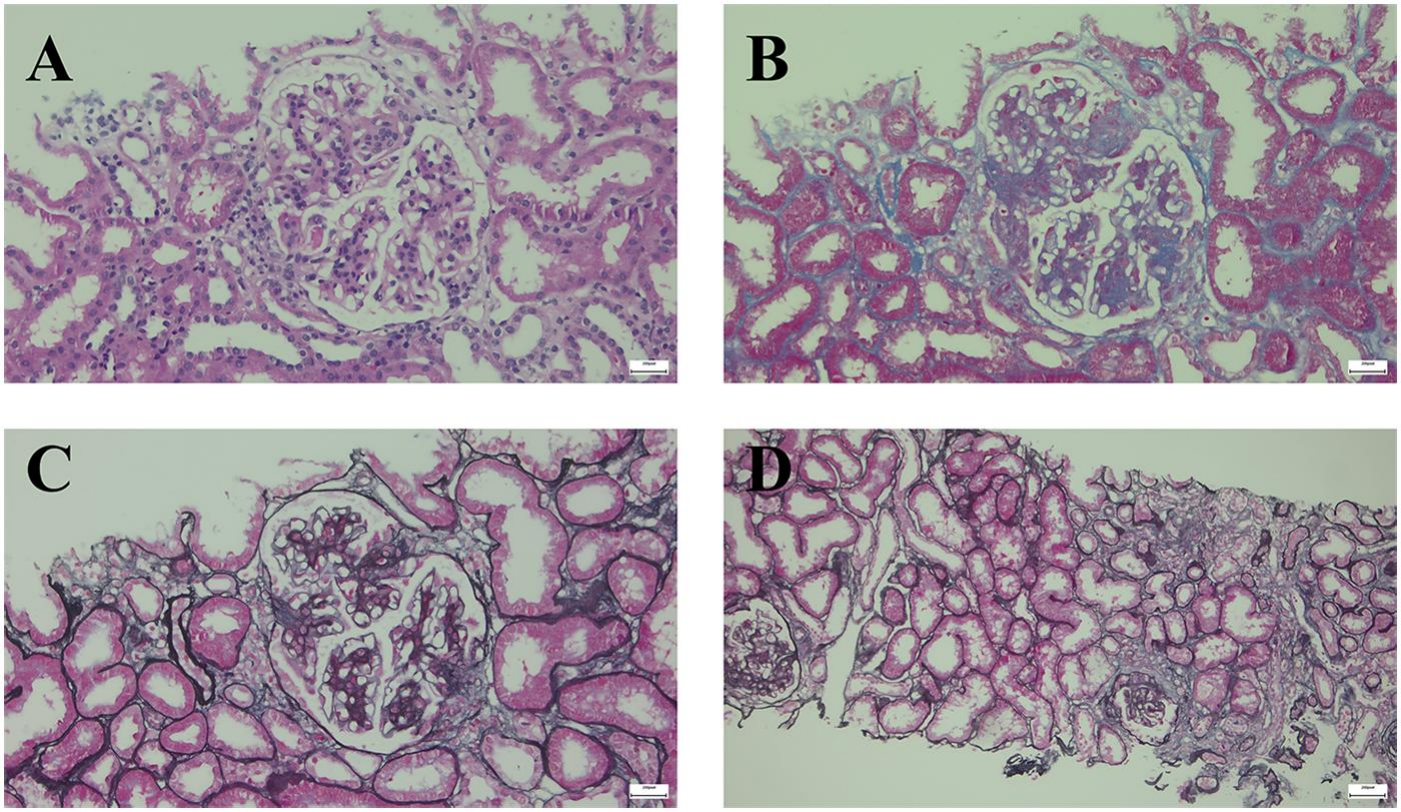
Light microscopy of renal biopsy: **(A)** H&E staining, **(B)** masson staining, and **(C)** PASM staining: moderate to severe hyperplasia of mesangium cells and stroma, and small cellular fibrous crescent formations with glomerular and bowman's capsule adhesions; **(D)** PASM staining: renal tubular epithelial cells displayed granular degeneration, with slight focal atrophy, focal lymphoid and mononuclear cell infiltration with fibrosis in the renal interstitium.

Renal biopsy revealed the following: (1) light microscopy (Figure 1): 17 glomerulus, diffuse moderate to severe hyperplasia of mesangium cells and stroma, segmental heavy hyperplasia with sclerosis, and 4 small cellular fibrous crescent formations with glomerular and Bowman's capsule adhesions; renal tubular epithelial cells displayed vacuolar and granular degeneration, with slight focal atrophy; focal lymphoid and mononuclear cell infiltration with fibrosis in the renal interstitium; (2) immunofluorescence: five glomeruli; IgA+++ granular deposition in the mesangium; IgG+, IgM++, C3+, C4+, C1q+, FRA++ granular deposition in the segmental mesangium and capillary loops; (3) electron microscopy: one glomerulus was detected, the mesangial cells and matrix proliferated, high-density electron dense deposits were observed in the mesangial area; and the epithelial cells show foot processes fusion. The combination of clinical and renal biopsy findings was consistent with IgAN with the Oxford typing of M1E0S1T1.

The patient received pulse of 750 mg of methylprednisolone sodium succinate for 3 days, after which 0.5 g of mycophenolate mofetil (MMF) twice a day was added. After treatment, she left the hospital with an improved condition and normal urine. She was treated with oral prednisone 50 mg/day and MMF, and had received Nusinersen every 4 months after discharge from the hospital. After the situation stabilized, the prednisone was gradually reduced and discontinued on September 30, 2023. The last follow-up was August 17, 2024. Her general condition was good, and routine blood tests revealed no obvious abnormalities; urine routine: protein **±**, erythrocyte **±**; urine protein quantitative: 0.29 g/24 h; and cardiac enzymes: CK: 357 U/L, CKMB: 5.76 ng/L.

## 3 Discussion and conclusions

SMA is caused by biallelic (homozygous or compound heterozygous) loss-of-function variants in *SMN1*, a motor neuron gene located in region 5q13, leading to markedly reduced level of survival motor neuron (SMN) protein. *SMN2* is a nearly identical copy of *SMN1*, due to a critical nucleotide difference in exon 7, most *SMN2* transcripts undergo exon 7 skipping, producing an unstable truncated protein (SMN*Δ*7) that is essentially nonfunctional and rapidly degraded (5, 6).

SMN proteins are widely expressed in the human body, and recent studies have shown that SMA may not only be a motor neuron disease but also affect multiple systems. The kidney is one of the organs affected (7–9). To the best of our knowledge, no relevant reports on SMA with IgAN have been found in the literature at present. West et al. (2) reported that a patient with SMA type 3 was found to have mild renal insufficiency and proteinuria, and a renal biopsy revealed microscopic lesions. Thomson et al. (4) encountered a patient with Kugelberg-Welander disease (SMA type 3) who presented with Hodgkin's disease complicating nephrotic syndrome after many years, and his renal biopsy was consistent with minimal glomerular abnormalities. Notably, Qian et al. (7) found that SMN levels in renal tubular cells were significantly reduced in biopsy samples from patients diagnosed with ischemic acute kidney injury (AKI) and in the mouse model of ischemia-reperfusion injury (IRI), that the degree of reduced SMN expression was correlated with the degree of renal impairment, and that the NF-κB signaling pathway may be involved in the process of aggravation of renal IRI due to SMN insufficiency. Nery et al. (10) suggested that patients with SMA type 1 showed altered concentrations of serum creatinine, cystatin C, sodium, glucose, and calcium, and other renal manifestations included tubular damage, nephrocalcinosis, and fibrosis. The latest large cohort study from the Netherlands indicates that patients with SMA are at risk of impaired renal function, which does not improve after 1–2 years treatment with *SMN2*-splicing modifying therapies (11). These studies suggest that there may be some interactions between SMN and renal function, but the exact mechanism needs to be further investigated.

A noteworthy point is that the patient in this case presented with obesity. Existing research indicates that obesity exerts a certain influence on the progression of IgAN. In conjunction with previous reports, we propose that SMA itself may constitute a distinct predisposing background—widespread deficiency of the SMN protein may lead to abnormalities in immune regulatory functions. This underlying state could render patients more susceptible to renal immune complex deposition when exposed to additional stimuli, such as obesity-related metabolic stress.

This patient received nusinersen sodium treatment after being diagnosed with SMA. Nusinersen is an antisense oligonucleotide (ASO) drug that promotes increased production of full-length SMN proteins by modifying the splicing of *SMN2* pre-messenger RNA (12), and the efficacy and safety of this drug have been demonstrated in pediatric SMA patients (13). Patients receiving long-term treatment with ASO drugs may develop proteinuria, which may be related to the accumulation of ASO drugs in the proximal renal tubules, thereby affecting their reabsorption of protein. It presents an opportunity to reflect on its potential impact on renal function. On the one hand, elevating SMN protein levels may theoretically enhance its potential protective effects within renal cells; on the other hand, the patient's urine abnormalities were present prior to the treatment, suggesting her IgAN is not considered drug-related. Consequently, this case underscores a critical clinical issue: establishing long-term, systematic monitoring of renal function and urine parameters is paramount for SMA patients undergoing SMN-upregulating therapies. This not only facilitates early detection of potential coexisting renal disease but also enables future data accumulation to clarify the long-term impact of the treatment on renal function in SMA patients—whether it confers potential protective effects, remains neutral, or carries risks. This introduces a new dimension to the multidisciplinary management of SMA. Current evidence linking ASO therapies, including nusinersen, to clinically significant chronic kidney disease remains limited; therefore, careful longitudinal monitoring and systematic data collection in SMA cohorts are essential to clarify this potential risk.

This case highlights that in the comprehensive management of SMA patients throughout their disease course, attention should be paid to monitoring other systems besides the neuromuscular system, such as renal involvement. However, considering that IgA nephropathy is one of the most common primary glomerular diseases worldwide, its coexistence with SMA may also be purely coincidental. Therefore, more clinical cases and long-term follow-up data are needed to confirm any potential relationship between SMA and IgAN, with the ultimate goal of optimizing diagnostic and therapeutic strategies for affected patients.

## Data Availability

The original contributions presented in the study are included in the article/Supplementary Material, further inquiries can be directed to the corresponding authors.
